# Comparison of Volatile Organic Compounds of *Sideritis romana* L. and *Sideritis montana* L. from Croatia

**DOI:** 10.3390/molecules26195968

**Published:** 2021-10-01

**Authors:** Tihana Marić, Maja Friščić, Zvonimir Marijanović, Željan Maleš, Igor Jerković

**Affiliations:** 1 Department of Pharmaceutical Botany, University of Zagreb Faculty of Pharmacy and Biochemistry, Schrottova 39, 10 000 Zagreb, Croatia; tihana.vilovic@pharma.unizg.hr (T.M.); zeljan.males@pharma.unizg.hr (Ž.M.); 2 Department of Food Technology and Biotechnology, Faculty of Chemistry and Technology, University of Split, Ruđera Boškovića 35, 21 000 Split, Croatia; zmarijanovic@ktf-split.hr; 3 Department of Organic Chemistry, Faculty of Chemistry and Technology, University of Split, Ruđera Boškovića 35, 21 000 Split, Croatia

**Keywords:** *Sideritis romana*, *Sideritis montana*, terpenes, HS-SPME, GC-MS, bicyclogermacrene, germacrene D, viridiflorol, principal component analysis

## Abstract

A study on the headspace volatile organic compounds (VOCs) profile of native populations of *Sideritis romana* L. and *Sidertis montana* L., Lamiaceae, from Croatia is reported herein, to elucidate the phytochemical composition of taxa from this plant genus, well-known for traditional use in countries of the Mediterranean and the Balkan region. Headspace solid-phase microextraction (HS-SPME), using divinylbenzene/carboxene/polydimethylsiloxane (DVB/CAR/PDMS) or polydimethylsiloxane/divinylbenzene (PDMS/DVB) fiber, coupled with gas chromatography-mass spectrometry (GC-MS) was applied to analyze the dried aerial parts of six native populations in total. Furthermore, principal component analysis (PCA) was conducted on the volatile constituents with an average relative percentage ≥1.0% in at least one of the samples. Clear separation between the two species was obtained using both fiber types. The VOCs profile for all investigated populations was characterized by sesquiterpene hydrocarbons, followed by monoterpene hydrocarbons, except for one population of *S. romana*, in which monoterpene hydrocarbons predominated. To our knowledge, this is the first report on the VOCs composition of natural populations of *S. romana* and *S. montana* from Croatia as well as the first reported HS-SPME/GC-MS analysis of *S. romana* and *S. montana* worldwide.

## 1. Introduction

Essential oils (EOs) are natural, volatile and complex mixtures of lipophilic compounds, often including terpenes, phenol-derived aromatic compounds and aliphatic compounds, that are biosynthesized by aromatic plants as their secondary metabolites and represented by a strong odor [1]. Ecologically, they are important for the plant defense system by providing protection against grazers, taking part in fire and drought tolerance and attracting pollinators and other animals for seed dispersal. The most often reported bioactivities of EOs and their components are antimicrobial, antiviral, antinociceptive, anticancer, anti-inflammatory, digestive, semiochemical and free radical scavenging [2], while new potential applications in human health, nutrition, agriculture and the environment are continuously emerging [1].

The volatile components of EOs are biosynthesized in specialized secretory structures such as glandular trichomes located on the surface, or secretory cells and ducts situated within the tissues of various plant organs of aromatic plants. The conventional techniques for volatile organic compounds (VOCs) extraction include, e.g., hydrodistillation and steam distillation, expression (cold pressing), solvent extraction [3] and *enfleurage* (a classical method for extracting volatile oil from flowers using a layer of animal fat) [4]. Along with conventional methods used for the extraction of volatile compounds from plants, solid-phase microextraction (SPME) is considered a convenient alternative technique for a variety of sample matrices (liquid/solid samples or headspace (HS) vapors). This technique bypasses the distillation or solvent extraction procedure [2]. It uses a fused silica fiber that is externally coated with an appropriate stationary phase such as polydimethylsiloxane (PDMS), polyacrylate (PA), PDMS/divinylbenzene (DVB), carbowax (CW)/DVB, and carboxene (CAR)/PDMS on which the headspace VOCs are adsorbed [5]. After the extraction, the fiber is usually directly inserted into the injector for gas chromatography analysis usually coupled with mass spectrometry (GC-MS), which is the most often used technique for the chemical characterization of EOs [2]. SPME has been widely used for chemical analysis of volatile components of different types of food substances, flavors, and medicinal plant materials because it is a simple, rapid, inexpensive and solvent-free technique that demands only a small amount of sample and reduces the degradation of VOCs [6,7]. Furthermore, headspace solid-phase microextraction (HS-SPME) requires no sample pre-treatment, allows high selectivity for small compounds and reusability of various commercially available SPME fibers [8], which makes this technique generally well accepted for VOCs extraction.

The genus *Sideritis* L. (family Lamiaceae) includes more than 150 species of annual or perennial herbs and shrubs, distributed in temperate and tropical regions of the Northern Hemisphere, with the majority of species found in the Mediterranean area, North Africa, the Iberian Peninsula, the Middle East and in the Macaronesian region [9,10,11]. The use of aerial parts of many *Sideritis* species has been well reported, especially in Albania, Bulgaria, Greece, Macedonia and Turkey, where they are consumed as herbal teas and applied in traditional medicine for the treatment of various disorders of the gastrointestinal and respiratory system, as well as for burns and wounds [10,12].

In recent decades, many researchers have been trying to justify the traditional uses of *Sideritis* species by elucidating their phytochemical composition and investigating potentially useful pharmacological activities of these species [10,13,14]. Although species from Lamiaceae family are well known for their aromatic properties and are recognized as the most important source of EOs with economical interest among Angiosperms [3], the species of the genus *Sideritis* have been reported as poor in EOs [10,15,16]. Nevertheless, for the pharmacological activity of *Sideritis* species, terpenes and flavonoids seem to be the constituents that are most important. Other chemical components which have been identified within the genus are iridoids, coumarins, lignans, phenylpropanoid glycosides and sterols [15].

Only three taxa of this genus are reported to be native to Croatia: *Sideritis romana* L., *S. romana* L. subsp. *purpurea* (Talbot ex Benth.) Heywood and *S. montana* L. [17]. While several reports on EOs of *S. romana* L. [18,19], *S. romana* subsp. *purpurea* [20,21] and *S. montana* L. [18,22,23,24,25,26] isolated by hydrodistillation have already been published, as well as that of *S. romana* subsp. *purpurea* EO constituents obtained by steam distillation [27], the composition of the headspace volatile components extracted using HS-SPME has never been evaluated for these species. Furthermore, to the best of our knowledge, the only report on using the HS-SPME technique for the detection of volatile components within the genus *Sideritis* has been published for *S. ozturkii* Aytac and Aksoy [28]. Additionally, the volatile HS profiles of *S. scardica* Griseb. and *S. raeseri* Boiss. and Heldr. were analyzed using GC-FID/MS [29], while EO composition of *S. albiflora* Hub.-Mor. was analyzed by both HS GC-MS and thermal desorption GC-MS [30].

The aim of the present study was to compare the VOCs composition of the aerial parts of *S. romana* L. and *S. montana* L. populations from different parts of Croatia and to determine characteristic volatile compounds for each species. Having in mind that the composition of volatile compounds may vary depending on environmental conditions [31,32], in comparison to similar studies conducted on hydrodistilled EOs of these species, which were based on the analysis of single populations [18,19,22,23,24,25,26], the advantage of our study is a greater number of investigated *S. romana* (four) and *S. montana* (two) populations. Considering that, on one hand, *Sideritis* species are generally poor in essential oil, and the limited availability of plant material in natural habitats from which the samples had been collected, and, on the other hand, the multiple advantages of HS-SPME, which have been mentioned above, instead of the conventionally used hydrodistillation, the latter technique was chosen for the isolation of volatile components, which were further analyzed by using GC-MS, with the aim to obtain more reliable results. Previous studies have shown that hydrodistillation and HS-SPME can detect similar major volatile constituents of individual plant species, although their relative percentages may vary depending on the technique used [33]. Having in mind that HS-SPME may, among other things, depend on the type of fiber used for extraction [34], the volatile components were extracted by HS-SPME using two different fibers suitable for untargeted analysis [35], divinylbenzene/carboxene/polydimethylsiloxane (DVB/CAR/PDMS) and polydimethylsiloxane/divinylbenzene (PDMS/DVB) fiber. To account for possible genetically and/or ecologically dependent variabilities in VOCs composition, several populations of *S. romana* (four populations) and *S. montana* (two populations) have been included in the analysis and principal component analysis (PCA) was conducted on the major volatile constituents (average relative percentage ≥ 1.0% in at least one of the samples) obtained by each fiber to examine the interrelationships among the investigated populations. Furthermore, the obtained results have been compared to previous findings for the same two species that were mostly based on hydrodistillation. Finally, the VOCs profile was compared to related species and discussed considering the reported biological activities of major constituents. To our knowledge, this study is the first report on the VOCs profile of natural populations of *S. romana* and *S. montana* from Croatia.

## 2. Results

### 2.1. HS-SPME/GC-MS Analysis

#### 2.1.1. DVB/CAR/PDMS Fiber

**Figure 1:**
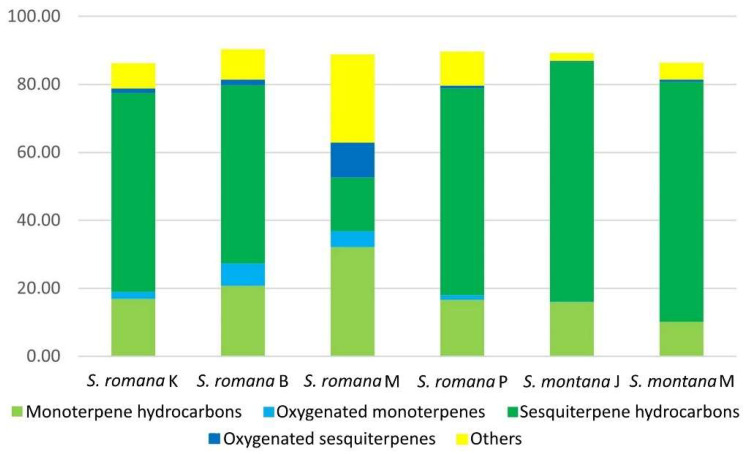
Average relative percentages of different classes of headspace volatile organic compounds obtained from various populations of *S. romana* and *S. montana* using DVB/CAR/PDMS fiber.

VOCs of four populations of *Sideritis romana* and two populations of *S. montana* were extracted by HS-SPME, using DVB/CAR/PDMS fiber and their composition was analyzed by GC-MS. In total, 69 VOCs were identified, accounting for 86.18–90.29% of the total VOCs content (Table 1). The VOCs compositions of investigated populations of both investigated species were characterized by sesquiterpene hydrocarbons, followed by monoterpene hydrocarbons, except for that of *S. romana* from Morinje Bay (*S. romana* M), in which monoterpene hydrocarbons and other compounds were more abundant than sesquiterpene hydrocarbons. Additionally, for the latter, oxygenated sesquiterpenes were more represented than in other investigated samples (Figure 1). The main individual compounds that were identified for *S. romana* were bicyclogermacrene (15.16–23.04%) (except *S. romana* M), *trans*-caryophyllene (3.60–23.88%) (except *S. romana* from Kamenjak (*S. romana* K) and Blato (*S. romana* B)), *trans*-*β*-farnesene (1.00–12.79%), limonene (1.68–7.58%), alloaromadendrene (2.35–7.32%), *β*-pinene (2.61–8.01%), and isocaryophyllene (2.39–4.70%) (except *S. romana* M). Exceptionally, for *S. romana* M, the main identified compounds were benzyl alcohol (13.28%), viridiflorol (10.29%), ledene (8.20%), limonene (7.10%), *β*-pinene (6.67%), and *p*-cymene (5.49%). For *S. montana*, the main identified individual compounds were germacrene D (17.04–23.23%), *trans*-caryophyllene (6.56–11.89%), *δ*-cadinene (7.57–8.85%), bicyclogermacrene (4.19–11.76%), limonene (6.31–8.14%), and *trans*-*β*-farnesene (3.56–4.83%) (Table 1).

#### 2.1.2. PDMS/DVB Fiber

**Figure 2:**
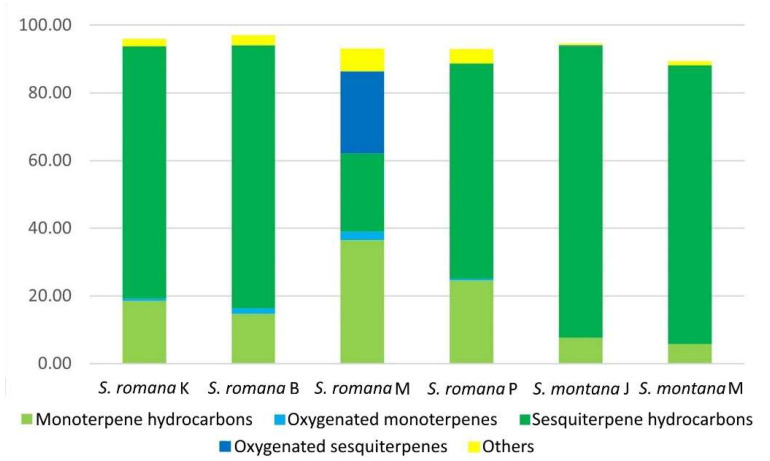
Average relative percentages of different classes of headspace volatile organic compounds obtained from various populations of *S. romana* and *S. montana* using PDMS/DVB fiber.

VOCs of the same four populations of *Sideritis romana* and two populations of *S. montana* were extracted by HS-SPME, using also PDMS/DVB fiber and their composition was analyzed by GC-MS. In total, 56 VOCs were identified, accounting for 89.35–97.09% of the total VOCs content (Table 2). As with DVB/CAR/PDMS fiber, the VOCs compositions of investigated populations of both species were characterized by sesquiterpene hydrocarbons, followed by monoterpene hydrocarbons, except for that of *S. romana* M, in which monoterpene hydrocarbons and oxygenated sesquiterpenes were more abundant than sesquiterpene hydrocarbons. Moreover, other compounds were more abundant in this population than in other investigated samples (Figure 2). By applying PDMS/DVB fiber, more sesquiterpene hydrocarbons, and less oxygenated monoterpenes and other compounds were extracted than with the application of DVB/CAR/PDMS fiber. The main individual compounds that were identified for *S. romana* were bicyclogermacrene (10.21–57.50%), *β*-pinene (6.78–13.26%), isocaryophyllene (5.17–7.10%) (except *S. romana* M), *trans*-*β*-farnesene (3.55–7.71%), germacrene D (1.22–12.18%), *γ*-terpinene (0.11-8.96%), limonene (1.97–3.86%) and *α*-pinene (2.09–3.91%). For *S. romana* M, the main individual compounds were viridiflorol (24.19%), *β*-pinene (13.26%), bicyclogermacrene (10.21%), *γ*-terpinene (8.96%) and *trans*-caryophyllene (6.35%). In *S. montana*, the main individual compounds were germacrene D (51.08–53.63%), bicyclogermacrene (9.54–18.03%), *trans*-caryophyllene (6.65–15.49%), limonene (2.91–3.74%) and *trans*-*β*-farnesene (2.17–4.02%) (Table 2).

### 2.2. Principal Component Analysis

In order to analyze the differences between VOCs compositions of investigated *S. romana* and *S. montana* populations, principal component analysis of the major identified components of both taxa was performed, including only those compounds showing at least 1.0% of the total VOCs content in at least one of the samples (38 compounds in total), i.e., 37 compounds identified using DVB/CAR/PDMS fiber and 17 compounds identified using PDMS/DVB fiber (Table 3).

#### 2.2.1. DVB/CAR/PDMS Fiber

**Figure 3:**
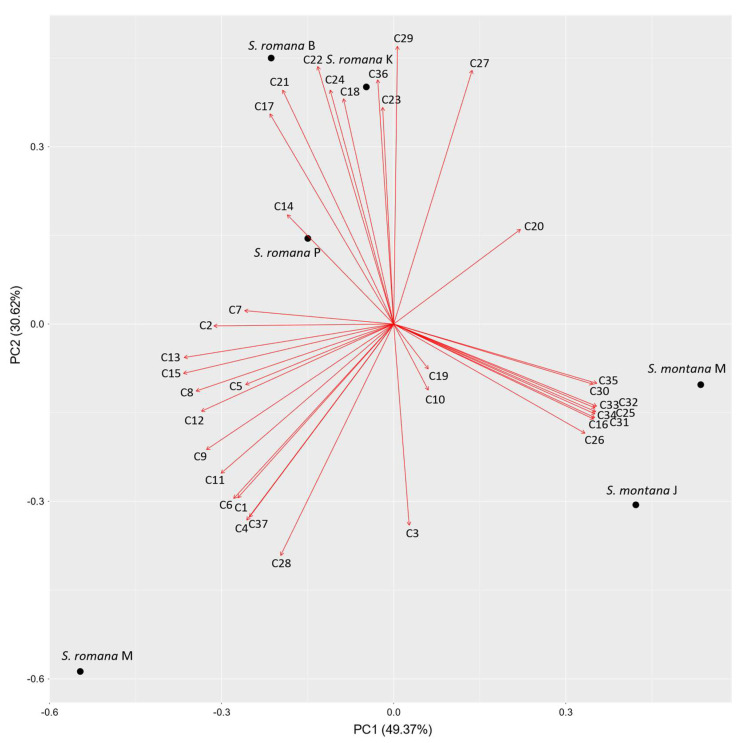
Biplot obtained by principal component analysis of VOCs composition of investigated populations of *S. romana* and *S. montana*, based on major components with average percentages ≥ 1% in at least one of the samples, detected with DVB/CAR/PDMS fiber.

The biplot constructed by the first two principal components that are showing the distribution of investigated *S. romana* and *S. montana* populations and VOCs identified with DVB/CAR/PDMS fiber is presented in Figure 3. Principal component 1 (PC1) accounted for 49.37% and principal component 2 (PC2) for 30.62% of total variance in the data. Clear separation between *S. romana* and *S. montana* was obtained. For all populations of *S. romana*, except for *S. romana* M, distinctive components for discrimination observed by using DVB/CAR/PDMS fiber were bicyclogermacrene (C29), alloaromadendrene (C21), *trans*-*β*-farnesene (C23), isocaryophyllene (C24), selina-5,11-diene (C22), *α*-gurjunene (C17) and spathulenol (C36), while *S. romana* M, was characterized by benzyl alcohol (C11), viridiflorol (C37), ledene (C28), *p*-cymene (C9), acetic acid (C1), benzaldehyde (C4) and *β*-myrcene (C6). Distinctive components for discrimination of *S. montana* populations were germacrene D (C26), *δ*-cadinene (C32), *γ*-cadinene (C31), *α*-amorphene (C25), *α*-muurolene (C30), *α*-copaene (C16), *α*-cadinene (C34), cadina-1,4-diene (C33) and *α*-calacorene (C35).

#### 2.2.2. PDMS/DVB Fiber

**Figure 4:**
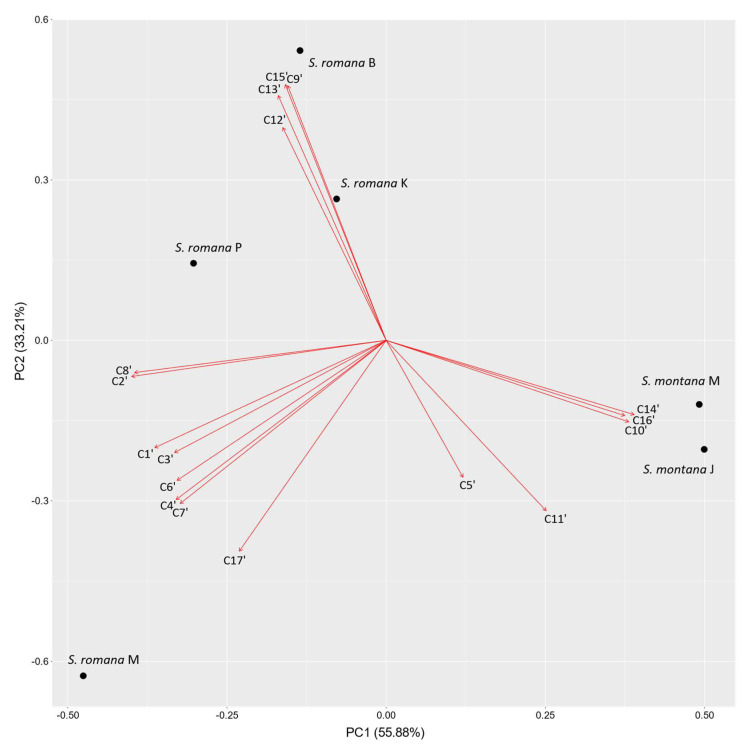
Biplot obtained by principal component analysis of VOCs composition of investigated populations of *S. romana* and *S. montana*, based on major components with average percentages ≥ 1% in at least one of the samples, detected with PDMS/DVB fiber.

The biplot constructed by the first two principal components that are showing the distribution of investigated *S. romana* and *S. montana* populations and VOCs identified with PDMS/DVB fiber is presented in Figure 4. Principal component 1 (PC1) accounted for 55.88% and principal component 2 (PC2) for 33.21% of total variance in the data. Clear separation between *S. romana* and *S. montana* was obtained. Distinctive components observed by using PDMS/DVB fiber for all *S. romana* populations, except for *S. romana* M, were bicyclogermacrene (C15′), isocaryophyllene (C13′), *trans*-*β*-farnesene (C12′) and bicycloelemene (C9′). *S. romana* M population was characterized by viridiflorol (C17′), *γ*-terpinene (C7′), *α*-pinene (C1′), *p*-cymene (C4′), benzyl alcohol (C6′) and *α*-terpinene (C3′). Distinctive components for discrimination of *S. montana* were germacrene D (C14′), *α*-copaene (C10′) and *δ*-cadinene (C16′).

## 3. Discussion

Depending on the polarity of VOCs of an analyte, several types of fibers may be used for extracting different groups of compounds such as PDMS (a non-polar fiber used for volatile compounds), PA (a polar fiber used for polar semi-volatile compounds), CW/DVB (a polar fiber used for alcohols and volatiles), PDMS/DVB (a bipolar fiber used for volatile compounds of medium polarity, amines and nitroaromatics), CAR/PDMS (a bipolar fiber used for low molecular weight volatile compounds) and DVB/CAR/PDMS (a bipolar fiber used for polar and non-polar volatile compounds) [34,36]. In this study, DVB/CAR/PDMS and PDMS/DVB fiber, which were previously shown to be most suitable for untargeted HS-SPME analysis of volatiles [35], were used for the analysis of VOCs from aerial parts of *S. romana* and *S. montana* from Croatia. In our study, the number of extracted VOCs was higher using DVB/CAR/PDMS fiber (69 compounds) than it was by using PDMS/DVB fiber (56 compounds), which is in accordance with the work of Wang et al. [37], in which DVB/CAR/PDMS fiber showed better efficiency to extract volatile compounds (50 compounds) from the samples of *Aquilegia japonica* Nakai and H.Hara, compared to CAR/PDMS (47 compounds) and PDMS/DVB fibers (45 compounds), as well as with the work of Sukkaew et al. [38], where more volatile components were extracted using DVB/CAR/PDMS fiber (51 compounds) than with PDMS/DVB (38 compounds), PDMS (38 compounds) and CAR/PDMS (37 compounds) fibers in *Murraya koenigii* (L.) Sprengel fresh leaves. In our study, the dominant chemical classes of volatile compounds in all samples of the two investigated *Sideritis* species extracted using DVB/CAR/PDMS and PDMS/DVB fibers were sesquiterpene hydrocarbons followed by monoterpene hydrocarbons, except for *S. romana* M in whose extract monoterpene hydrocarbons were more abundantly present. The obtained results for *S. montana* are in accordance with the results reported by Venditti et al. [23] for EO of *S. montana* subsp. *montana* from central Italy obtained by hydrodistillation, where sesquiterpene hydrocarbons led by germacrene D and bicyclogermacrene were observed as the most abundant EO components. According to the latter, oil-poor species from Lamioideae subfamily produce EOs rich in sesquiterpene hydrocarbons. Furthermore, our results are in accordance with the classification of *Sideritis* species from Turkey given by Kirimer et al. [18], based on the main components of hydrodistilled EOs, where *S. montana* ssp. *montana* was classified into the sesquiterpene hydrocarbons-rich group, and *S. romana* ssp. *romana* into the oxygenated monoterpenes-rich group. Considering the relative percentages of extracted chemical classes, in comparison to PDMS/DVB fiber, DVB/CAR/PDMS fiber extracted more oxygenated monoterpenes and other compounds (mostly non-terpenes), which were more abundantly present in investigated *S. romana* populations. Conversely, in accordance with previously reported findings [36], PDMS/DVB fiber was observed to be more efficient in extracting sesquiterpene hydrocarbons, which were relatively higher in investigated *S. montana* populations when comparing to the results obtained for the *S. romana* populations included in this study. In fact, for both species, the most abundant VOCs, namely the sesquiterpene hydrocarbons germacrene D (*S. montana*) and bicyclogermacrene (*S. romana*, except for *S. romana* M) were extracted more efficiently by PDMS/DVB fiber. The same was observed for viridiflorol, an oxygenated sesquiterpene that was found as the major characteristic compound of the chemically distinct population of *S. romana* from Morinje Bay (*S. romana* M).

The dominant compounds of *S. romana* (except *S. romana* M) extracted with DVB/CAR/PDMS fiber were bicyclogermacrene, *trans*-caryophyllene, *trans*-*β*-farnesene, limonene and alloaromadendrene, while bicyclogermacrene, *β*-pinene, isocaryophyllene, *trans*-*β*-farnesene, germacrene D and *γ*-terpinene were dominant after the extraction with PDMS/DVB fiber. A different composition of EO was reported for *S. romana* from Italy, where carvacrol, limonene and 1,8-cineole were reported as the dominant compounds isolated by hydrodistillation [19], while neither carvacrol and 1,8-cineole nor thymol, oct-1-en-3-ol and borneol, reported as the dominant compounds isolated by hydrodistillation from *S. romana* subsp. *romana* from Turkey [18], were detected in our samples of *S. romana* from Croatia. For *S. romana* M, the dominant compounds extracted with DVB/CAR/PDMS were benzyl alcohol, viridiflorol, ledene and limonene, while with PDMS/DVB these were viridiflorol, *β*-pinene, bicyclogermacrene and *γ*-terpinene. These results are not in accordance with the previously mentioned studies of *S. romana* from Italy and Turkey. For other subspecies of *S. romana*, such as *S. romana* subsp. *purpurea* from Greece, bicyclogermacrene, *β*-caryophyllene (=*trans*-caryophyllene or isocaryophyllene), *γ*-muurolene, *β*-pinene, (*E*)-*β*-farnesene (=*trans*-*β*-farnesene) and spathulenol were found as the major components obtained by hydrodistillation [21], while for the same subspecies from Montenegro, the major EOs constituents obtained by steam distillation were *γ*-elemene and spathulenol [27]. Moreover, the most abundant EO components obtained by hydrodistillation from the same subspecies, which was also collected in Montenegro, included bicyclogermacrene, germacrene D, (*E*)-caryophyllene and spathulenol [20]. According to the PCA analysis, even though it was present with more than 1% only in one of the analyzed samples, spathulenol appeared to be one of the compounds distinctive for *S. romana*. However, it was not found in *S. romana* M, which was observed to contain much lower amounts of bicyclogermacrene, which were detected only using PDMS/DVB fiber. However, spathulenol may be an artifact, having in mind that bicyclogermacrene is easily converted to spathulenol at room temperature [39]. The compound may also be formed during the process of hydrodistillation, as seen in the comparison of EO composition and VOCs composition of *S. scardica* samples prepared by other techniques of isolation such as solvent extraction and supercritical carbon dioxide extraction [40]. On the other hand, distinctive VOCs of *S. romana* M such as viridiflorol (detected in higher amount using PDMS/DVB fiber) and ledene (detected only using DVB/CAR/PDMS fiber) may be the result of bicyclogermacrene hydration and/or rearrangement [41,42]. According to Carvalho et al. [43], who studied the volatile fractions of *in natura*, fresh, and dried *Casearia sylvestris* var. *sylvestris* Sw. and var. *lingua* (Cambess.) Eichler leaves, viridiflorol is an artifact most likely formed from bicyclogermacrene, whose content increases during the drying process. As it can be seen from Table 4, the distinct sample of *S. romana* from Morinje Bay (*S. romana* M) was collected prior to other samples of the same species and, therefore, may have been more susceptible to transformations during processing and storage of plant material, having in mind that all collected samples were analyzed simultaneously. The sample from Morinje Bay also contained more other compounds (non-terpenes), which were mostly oxygenated compounds (alcohols, aldehydes and a carboxylic acid), indicating its possible oxidative degradation. Other compounds that were present and/or were more abundant in *S. romana* populations other than *S. romana* M, such as alloaromadendrene and aromadendrene, may also have been formed by isomerization from bicyclogermacrene [44] and could have produced spathulenol by further oxidation [43].

Comparable to our results, variations in bicyclogermacrene content were observed in aroma compounds isolated by hydrodistillation of dried aerial parts from different populations of *S. scardica* and *S. raeseri* from Macedonia [29]. Moreover, it was reported by Kirimer et al. [46] in their extensive study conducted on 50 *Sideritis* taxa from the section *Empedoclia* that viridiflorol was one of the major components isolated by hydrodistillation from *S. perfoliata* L. from Turkey, while the same compound was not observed for this species in a subsequent study done by the same authors conducted on only two species [47]. Additionally, viridiflorol was found as one of the major constituents of EO isolated by hydrodistillation from *S. montana* subsp. *montana* from Turkey together with germacrene D, bicyclogermacrene and (*E*)-*β*-farnesene [18]. In the present study, the predominant compounds for *S. montana* extracted with DVB/CAR/PDMS fiber were germacrene D, *trans*-caryophyllene, *δ*-cadinene, bicyclogermacrene, limonene and *trans*-*β*-farnesene, while with PDMS/DVB fiber germacrene D was followed by bicyclogermacrene, *trans*-caryophyllene, limonene and *trans*-*β*-farnesene. In investigated samples of *S. montana*, as well as in the samples of *S. romana* other than *S. romana* M, viridiflorol was found only in trace amounts (<1%), while ledene was observed only in *S. montana* from Ježević (*S. montana* J), the latter being the previously collected and thus older of the two investigated samples of *S. montana* (stored for a slightly longer period before GC-MS analysis). Considering that a significantly lower amount of bicyclogermacrene was detected in the same sample compared to *S. montana* from Mosor (*S. montana* M), ledene may have been formed during the drying and storage period from the aforementioned bicyclogermacrene. Our results considering the major VOCs of investigated populations of *S. montana* are in accordance with the results of previous studies from Bulgaria [22], Turkey [18], Serbia [25] and Italy [23], in which germacrene D predominated in the chemical composition of EOs isolated from aerial parts by hydrodistillation, usually being followed by bicyclogermacrene. Different major compounds were reported for *S. montana* from Iran, in which geraniol particularly predominated in the EO obtained by hydrodistillation from the flowering spikes of the plant [26], while it was not detected in our samples of *S. montana* aerial parts, as well as for *S. montana* subsp. *montana* from Turkey, in which *β*-caryophyllene, *α*-pinene and *β*-pinene were found to be the major compounds of the hydrodistilled EO obtained from aerial parts of the plant [24]. The present study also indicated that *S. montana*, unlike *S. romana*, is characterized by several sesquiterpene hydrocarbons having a cadalane skeleton (*δ*-cadinene, *γ*-cadinene, *α*-amorphene, *α*-muurolene, *α*-cadinene, and cadina-1,4-diene). Clear separation between *S. montana* and *S. romana* samples was observed in the PCA with both fiber types used, with *S. romana* M showing a distinct VOCs profile.

Observed differences in the chemical composition of investigated Croatian *Sideritis* species from previously published data on VOCs composition of aerial parts from samples of different geographical origins may be explained by different ecological conditions (Table 4). According to Köppen climate classification [45], the collection areas from which the populations included in the present study were sampled belong to different climates including the Mediterranean climate with hot summer (*S. romana* B, *S. romana* M), the Mediterranean climate with warm summer (*S. romana* from Pražnica (*S. romana* P)), the temperate humid climate with hot summer (*S. romana* K, *S. montana* M) and the temperate humid climate with warm summer (*S. montana* J). This might have party affected their VOCs production. As it is known, VOCs composition can change depending on the type of stimulus received from the environment [32]. For example, variation of VOCs content may have been caused by environmental stress such as drought stress, as it was detected in the study of different cultivars of *Thymus vulgaris* L., in which *α*-phellandrene, *o*-cymene, *γ*-terpinene and *β*-caryophyllene were recognized as the compounds included in drought stress adaptation [48]. These compounds were observed as one of the major identified VOCs in the present study. For example, *γ*-terpinene was found to be especially dominant in *S. romana* M. Additionally, individual VOCs production might have been affected by the plant species that grew in the vicinity of investigated specimens in each habitat (place of collection) [49,50]. Although this has not been recorded in the present study, insight into the diversity of plant species in the harvesting locations can be partially gained through floristic studies that were conducted in the areas that are overlapping with or are near to the harvesting locations [51,52,53,54,55,56,57,58,59].

Besides ecological conditions, the observed differences may also be explained by the applied methods of extraction, having in mind that none of the previously mentioned studies used the HS-SPME method for extraction of VOCs from *S. romana* and *S. montana*. Instead, as already mentioned above, most authors used hydrodistillation, with the exception of the study done by Garzolli et al. [27], in which steam distillation was used. A few comparative analyses of plant volatile compounds isolated by hydrodistillation and HS-SMPE were published. A study on *Myrtus communis* L. showed that the applied microextraction techniques (HS-SPME and HS-SDME) extracted compounds that were more volatile, such as *α*-pinene and limonene, in comparison to hydrodistillation, which, in turn, obtained higher peak areas for low volatile compounds [60]. Moreover, analysis of volatile compounds from fruits of *Seseli libanotis* (L.) W.D.J.Koch also showed that higher contents of low-boiling compounds such as sabinene, *β*-phellandrene, *α*-pinene, *β*-pinene, *β*-myrcene, *γ*-terpinene and *α*-phellandrene may be extracted by using HS-SPME than by hydrodistillation, while higher amounts of high-boiling compounds (higher molecular mass and lower volatility) were observed using hydrodistillation at high temperature [61]. Qualitative and quantitative differences of volatile compounds of *Melissa officinalis* L. were observed after applying hydrodistillation and HS-SPME, which may be attributed to the formation of artifacts [6]. Furthermore, variations in the percentages and nature of compounds adsorbed on a SPME fiber compared to those found in hydrodistilled EOs were reported for *Petroselinum crispum* Mill. [62], while the comparison of the same techniques on *Bupleurum plantagineum* Desf. showed differences mainly in minor components [63]. Therefore, despite some differences, the results obtained by HS-SPME considering the major VOCs may be comparable to the results obtained by hydrodistillation [61,62,63].

In the studied samples, the most abundant VOCs, depending on the fiber used for solid-phase microextraction, were bicyclogermacrene (*S. romana* K, *S. romana* B and *S. romana* P) and/or *trans*-caryophyllene (*S. romana* P), benzyl alcohol or viridiflorol (*S. romana* M), and germacrene D (*S. montana* J and *S. montana* M). These compounds were previously shown to possess certain biological activities that may be of interest to the pharmaceutical, food and/or cosmetics industry. For example, bicyclogermacrene displayed cytotoxic activity against a range of cancer cell lines (IC_50_ = 3.1–21 μg/mL [64] and IC_50_ = 1.5–4.4 μg/mL). The same was observed for germacrene D (IC_50_ = 2.7–8.0 μg/mL) [65]. Additionally, germacrene D and *trans*-caryophyllene were recognized as the major EO constituents of selected species from the genera *Siparuna* Aublet and *Piper* L. that possess antiradical activity [66]. According to Dahham et al. [67], *β*-caryophyllene possesses antibacterial activity against *S. aureus* (MIC = 3 ± 1.0 µM) as well as anti-fungal (MIC = 4–7 µM), antioxidant (IC_50_ = 1.25–3.23 µM), and anti-proliferative activity against colorectal cancer cells (IC_50_ = 19 µM). Benzyl alcohol is frequently used as a bacteriostatic agent in liquid pharmaceutical products [68] as well as a preservative in cosmetic products [69]. On the other hand, antiradical (IC_50_ = 57.55–74.7 μg/mL), anti-mycobacterial (MIC = 190.0 μg/mL) and anti-inflammatory activity (60% reduction of carrageenan-induced mice paw edema after 3–30 mg/kg oral administration) were reported for viridiflorol [70]. Moreover, viridiflorol was recently observed to induce intracellular Ca^2+^ mobilization in human neutrophils and C20 microglial cells [71].

Related species of the genus *Sideritis,* namely *S. scardica* [22,29,72], *S. clandestina* (Bory and Chaub.) Hayek [21], *S. raeseri* [72,73,74], and *S. syriaca* L. [22], whose aerial parts have been traditionally used for the treatment of inflammation, gastrointestinal disorders and cough associated with cold and whose usage has been approved by the European Medicines Agency (EMA) Committee on Herbal Medicinal Products (HMPC) [75], were observed to possess, as one of their major volatile constituents, the same VOCs that were found in *S. romana* and *S. montana* in the present study, thus, indicating a promising potential for future investigations of these species.

## 4. Materials and Methods

### 4.1. Plant Material

**Figure 5:**
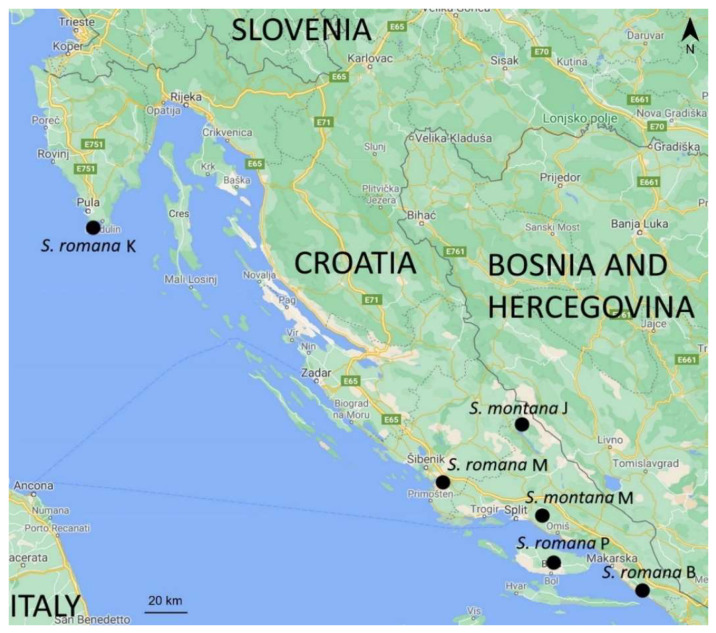
Harvesting locations for investigated *Sideritis* species.

Aerial parts of *S. romana* (four samples, consisting of 5–15 cm long shoots) and *S. montana* (two samples, consisting of 7–26 cm long shoots) were collected during the flowering period in June 2020 from natural populations on several locations in Croatia (Figure 5). In the field, the plant material was stored in paper bags and later spread out into one layer to dry out, in a place with room temperature. The mass of dried samples ranged between 2 and 22 g for *S. romana* and between 3 and 11 g for *S. montana*. Taxonomic identification was performed by T. M. Voucher specimens have been deposited within the Herbarium of the Department of Pharmaceutical Botany, University of Zagreb Faculty of Pharmacy and Biochemistry, Croatia. Sample codes, voucher numbers, harvesting locations and geographic coordinates are given in Table 4.

### 4.2. SPME Fibers and Extraction Procedure

HS-SPME was achieved with a manual SPME holder by using two fibers, (divinylbenzene/carboxene/polydimethylsiloxane (DVB/CAR/PDMS) and polydimethylsiloxane/divinylbenzene (PDMS/DVB)), that were conditioned according to Supelco Co. instructions before extraction. Cut samples (1 g) were placed separately in glass vials (5 mL) and sealed hermetically using PTFE/silicone septa. The vials were maintained in a water bath (60 °C) during equilibration (15 min) and extraction by HS-SPME (45 min). After extraction, the SPME fiber was withdrawn and inserted into the GC-MS injector (250 °C) for thermal desorption (6 min). The treatment was similar as previously reported [76]. HS-SPME was performed in duplicate and average values are presented in Table 1, Table 2 and Table 3.

### 4.3. GC-MS Analysis

GC-MS analyses were performed on a gas chromatograph model 7820A (Agilent Technologies, Palo Alto, CA, USA) containing a HP-5MS capillary column (5% phenyl-methylpolysiloxane, Agilent J and W; 30 m × 0.25 mm i.d., coating thickness 0.25 µm) and a mass selective detector (MSD) model 5977E (Agilent Technologies, Palo Alto, CA, USA). The GC conditions were described previously [76,77]. The carrier gas was helium (He 1.0 mL/min). The oven temperature was set at 70 °C for 2 min, then it was increased from 70 °C to 200 °C at a rate of 3 °C/min, and held at 200 °C for 15 min. The MSD (EI mode) was used at 70 eV, and 30–300 amu mass range was applied.

The compounds identification was based on the retention indices (RIs) determined relative to retention times of *n*-alkanes (C_9_-C_25_) and their comparison with literature data (National Institute of Standards and Technology) as well as by their mass spectra compared with the spectra from Wiley 9 (Wiley, New York, NY, USA) and NIST 17 (Gaithersburg, MD, USA) mass spectral libraries. The percentage composition was calculated using the normalization method (without correction factors). The average component percentages in Table 1–3 were calculated from duplicate GC-MS analyses [76,77].

### 4.4. Principal Component Analysis

Principal component analysis (PCA) was conducted on the volatile constituents having an average relative percentage ≥ 1.0% in at least one of the samples (in total, six observation of 37 variables for DVB/CAR/PDMS fiber and six observations of 17 variables for PDMS/DVB fiber), in order to examine the interrelationships among the investigated populations of *S. romana* (four populations) and *S. montana* (two populations), in RStudio version 1.4.1717 [78] using the function prcomp in R version 4.1.1. [79]. Scaling was set to “TRUE” to perform the analysis on normalized data. Plotting was performed using the function autoplot from the package ggfortify [80,81].

## 5. Conclusions

Headspace volatile organic compounds (VOCs) profile of aerial parts of altogether six native populations of *Sideritis romana* and *S. montana* from Croatia were determined by HS-SPME/GC-MS, using DVB/CAR/PDMS and PDMS/DVB fibers for compound extraction, which resulted in identification of 69 and 56 compounds, respectively. The most abundant VOCs in both species were those belonging to the class of sesquiterpene hydrocarbons, which were extracted more efficiently using PDMS/DVB fiber. The performed PCA analyses highlighted the major volatile constituents for each population and revealed clear separation between the investigated species using both fiber types. The most abundant VOCs found in the analyzed samples of *S. romana* were bicyclogermacrene and/or *trans*-caryophyllene or viridiflorol and benzyl alcohol, while germacrene D was the most abundant VOC found in the analyzed samples of *S. montana*, all of which were previously reported to possess certain biological activities that are potentially interesting for the pharmaceutical, food and/or cosmetics industry. The observed differences in the presence and amount of detected volatile compounds among the researched populations of the same species may be a result of different environmental and ecological conditions existing on the collection sites, while specific differences may also be attributed to changes that might have occurred during drying and storage of sampled plant material. The obtained results, according to which *S. romana* and *S. montana* show a promising potential for future utilization, suggest that the origin of plant material and/or growing as well as processing conditions should be considered as possible factors affecting VOCs production if these species were to be exploited.

## Figures and Tables

**Table 1:** Volatile organic compounds (VOCs) composition (%) of *S. romana* and *S. montana* obtained by HS-SPME/GC-MS with DVB/CAR/PDMS fiber.

No.	Compound	CD ^1^	RI ^2^	*S. romana* K	*S. romana* B	*S. romana* M	*S. romana* P	*S. montana* J	*S. montana* M
1	Acetic acid	C1	<900	-	0.85	3.11	1.33	0.32	0.55
2	Pentanal		<900	0.15	0.40	-	-	-	-
3	Hexanal		<900	0.07	0.08	0.52	0.24	0.02	0.04
4	(*E*)-Hex-2-enal		<900	0.24	0.13	0.90	0.42	0.14	0.47
5	*α*-Thujene	C2	936	0.47	1.03	1.08	0.21	0.16	0.05
6	*α*-Pinene	C3	944	1.32	0.94	2.51	2.19	3.42	1.23
7	Camphene		960	0.07	0.07	0.01	0.19	0.07	-
8	Benzaldehyde	C4	969	0.44	0.32	2.58	0.71	0.37	0.42
9	Hexanoic acid		975	0.12	-	-	-	-	0.09
10	Sabinene		981	0.27	0.44	0.21	0.09	0.13	0.09
11	Oct-1-en-3-ol		983	0.60	-	-	-	0.13	0.46
12	*β*-Pinene	C5	985	3.69	2.61	6.67	8.01	2.81	1.52
13	*β*-Myrcene	C6	995	0.80	0.84	1.27	0.88	0.91	0.64
14	(*E*,*E*)-Hepta-2,4-dienal		1001	-	0.07	0.63	-	0.05	-
15	*α*-Phellandrene	C7	1011	0.32	3.37	2.54	0.20	0.10	0.09
16	*α*-Terpinene	C8	1023	0.62	0.61	1.32	0.38	0.07	0.07
17	*p*-Cymene	C9	1032	0.99	1.81	5.49	1.32	0.04	0.05
18	Limonene	C10	1036	6.34	7.58	7.10	1.68	8.14	6.31
19	Benzyl alcohol	C11	1040	2.96	3.82	13.28	3.84	0.84	2.14
20	Phenylacetaldehyde		1051	0.12	-	-	-	-	0.09
21	*γ*-Terpinene	C12	1066	1.75	0.98	3.67	1.40	0.08	0.08
22	Octan-1-ol	C13	1075	1.37	2.22	3.55	2.50	0.35	0.29
23	*α*-Terpinolene		1093	0.33	0.52	0.34	0.05	0.08	-
24	Linalool		1103	-	0.26	-	-	-	-
25	Nonanal		1107	0.11	0.07	0.42	0.19	-	0.09
26	Benzeneethanol		1117	0.17	0.13	-	-	0.04	-
27	*α*-Campholenal		1132	0.07	-	-	-	-	-
28	*trans*-Pinocarveol ^3^		1145	-	0.36	0.77	0.40	-	-
29	Pinocarvone		1169	0.20	0.23	0.71	0.31	-	-
30	4-Terpineol		1182	0.60	0.95	0.89	0.15	0.05	0.07
31	Cryptone		1191	-	0.20	-	-	-	-
32	*α*-Terpineol	C14	1195	0.50	3.85	1.25	0.02	0.04	-
33	Myrtenal	C15	1199	0.39	0.54	1.03	0.47	-	-
34	Decanal		1208	0.16	0.10	-	0.23	-	-
35	Verbenone		1212	0.11	0.15	-	-	-	-
36	2-Phenoxyethanol		1223	0.15	0.06	-	-	-	-
37	Carvone		1249	0.12	-	-	-	-	-
38	Bicycloelemene		1342	0.75	0.53	-	0.40	0.22	0.41
39	*α*-Cubebene		1353	0.12	-	-	-	0.56	0.56
40	*α*-Copaene	C16	1375	0.18	-	-	-	1.67	2.02
41	Isoledene		1377	0.37	0.29	-	0.23	0.34	-
42	Longifolene		1395	0.56	0.34	-	0.28	0.37	-
43	*α*-Gurjunene	C17	1412	1.17	0.95	0.61	0.85	0.12	0.41
44	Aristolene	C18	1422	1.08	0.91	-	-	-	-
45	*trans*-Caryophyllene	C19	1423	-	-	3.60	23.88	11.89	6.56
46	Calarene		1436	0.33	0.31	-	0.34	-	0.19
47	Aromadendrene	C20	1438	1.11	1.05	-	0.96	0.23	2.87
48	Alloaromadendrene	C21	1443	7.32	6.69	2.35	6.27	0.76	0.90
49	Selina-5,11-diene ^3^	C22	1447	2.52	2.28	-	1.91	-	-
50	*γ*-Muurolene		1455	0.99	0.71	-	0.15	-	-
51	*trans*-*β*-Farnesene	C23	1461	5.12	12.79	1.00	5.58	4.83	3.56
52	Isocaryophyllene	C24	1471	4.70	2.39	-	3.22	-	-
53	*γ*-Gurjunene		1476	0.93	0.62	-	0.45	-	-
54	*α*-Amorphene	C25	1480	0.58	-	-	-	2.84	3.31
55	Germacrene D	C26	1484	1.50	0.06	-	-	23.23	17.04
56	trans-*β*-Ionone		1489	0.31	0.18	0.37	0.22	-	-
57	*β*-Cadinene	C27	1494	2.19	1.68	-	1.24	0.98	1.47
58	Ledene	C28	1497	-	-	8.20	-	2.27	-
59	Bicyclogermacrene	C29	1498	23.04	20.48	-	15.16	4.19	11.76
60	*α*-Muurolene	C30	1502	0.61	0.18	-	-	1.78	2.93
61	*γ*-Cadinene	C31	1517	0.58	-	-	-	3.33	3.37
62	*δ*-Cadinene	C32	1527	1.84	0.15	-	-	7.57	8.85
63	Dihydroactinidiolide		1532	0.41	0.41	0.60	0.33	0.11	0.35
64	Cadina-1,4-diene	C33	1536	0.20	-	-	-	0.94	1.24
65	*α*-Cadinene	C34	1541	0.24	-	-	-	1.41	1.70
66	*α*-Calacorene	C35	1547	0.47	0.04	-	-	1.05	1.36
67	Spathulenol	C36	1580	0.58	1.30	-	0.52	0.15	0.47
68	Viridiflorol	C37	1595	0.42	0.36	10.29	0.20	0.08	0.21
69	*α*-Cadinol		1658	0.34	-	-	-	-	-
Total identified [%]				86.18	90.29	88.87	89.60	89.28	86.38

^1^ CD: Compound designation of the major components (average percentages ≥ 1.0% in at least one of the samples), which were included in the principal component analysis. ^2^ RI: Retention index determined relative to a homologous series of *n*-alkanes (C_9_–C_25_) on a HP-5MS column; ^3^ Tentatively identified.

**Table 2:** Volatile organic compounds (VOCs) composition (%) of *S. romana* and *S. montana* obtained by HS-SPME/GC-MS with PDMS/DVB fiber.

No.	Compound	CD ^1^	RI ^2^	*S. romana* K	*S. romana* B	*S. romana* M	*S. romana* P	*S. montana* J	*S. montana* M
1	Pentanal		<900	0.02	-	-	0.03	-	-
2	Hexanal		<900	0.02	0.03	0.12	0.10	0.01	0.02
3	(*E*)-Hex-2-enal		<900	0.02	0.03	0.10	0.06	0.03	0.05
4	*α*-Thujene		936	0.55	0.41	0.79	0.58	0.07	0.04
5	*α*-Pinene	C1′	944	2.09	2.31	3.91	2.68	1.69	1.14
6	Camphene		960	0.05	0.04	0.10	0.06	0.02	-
7	Benzaldehyde		969	0.06	0.08	0.40	0.15	0.04	0.10
8	Sabinene		981	0.56	0.35	0.46	0.43	0.12	0.10
9	*β*-Pinene	C2′	985	6.78	8.01	13.26	10.35	1.80	1.46
10	*β*-Myrcene		995	0.55	0.40	0.99	0.80	0.28	0.20
11	(*E*,*E*)-Hepta-2,4-dienal		1001	-	0.05	0.12	0.07	0.02	0.03
12	*α*-Phellandrene		1011	0.15	0.83	0.27	0.22	0.02	-
13	*α*-Terpinene	C3′	1023	0.53	-	1.15	1.01	-	-
14	*p*-Cymene	C4′	1032	0.81	0.16	3.10	1.57	-	-
15	Limonene	C5′	1036	3.86	1.97	3.31	2.27	3.74	2.91
16	Benzyl alcohol	C6′	1040	0.63	0.96	2.55	1.12	0.16	0.55
17	(*Z*)-*β*-ocymene		1043	0.03	-	-	-	-	-
18	Phenylacetaldehyde		1051	0.03	-	-	-	-	0.02
19	(*E*)-*β*-ocymene		1054	0.02	-	0.11	-	-	-
20	*γ*-Terpinene	C7′	1066	2.53	0.11	8.96	4.49	-	-
21	Octan-1-ol	C8′	1075	1.17	1.33	2.46	2.24	0.12	0.16
22	*α*-Terpinolene		1093	0.11	0.07	0.19	0.14	0.01	-
23	Linalool		1103	-	0.86	0.25	0.22	-	-
24	Nonanal		1107	0.03	-	-	-	-	-
25	Benzeneethanol		1117	0.02	-	-	-	-	-
26	*trans*-Pinocarveol ^3^		1145	-	0.05	0.35	-	-	-
27	Pinocarvone		1169	0.10	0.04	0.26	-	-	-
28	4-Terpineol		1182	0.06	0.03	0.35	0.15	-	-
29	*α*-Terpineol		1195	0.22	0.58	0.41	-	-	-
30	Myrtenal		1199	0.15	0.07	0.70	0.11	0.02	-
31	Decanal		1208	-	-	0.19	0.11	-	-
32	Verbenone		1212	0.04	-	0.10	-	-	-
33	2-Phenoxyethanol		1223	0.01	-	-	-	-	-
34	Bicycloelemene	C9′	1342	2.18	2.40	0.43	1.73	0.34	0.68
35	*α*-Cubebene		1353	-	-	-	-	0.04	0.05
36	*α*-Copaene	C10′	1375	0.07	-	-	-	1.33	1.55
37	*α*-Gurjunene		1412	-	-	0.79	-	-	-
38	*trans*-Caryophyllene	C11′	1423	0.54	0.91	6.35	2.30	15.49	6.65
39	Aromadendrene		1438	0.25	-	-	-	-	-
40	Alloaromadendrene		1443	-	-	-	0.25	0.06	0.14
41	Selina-5,11-diene ^3^		1447	-	0.26	-	-	-	-
42	*γ*-Muurolene		1455	-	-	-	-	0.09	0.32
43	*trans*-*β*-Farnesene	C12′	1461	4.88	7.71	3.55	4.90	4.02	2.17
44	Isocaryophyllene	C13′	1471	7.10	6.85	-	5.17	-	-
45	*α*-Amorphene		1480	-	-	-	-	0.19	-
46	Germacrene D	C14′	1484	12.18	2.16	1.77	1.22	53.63	51.08
47	*trans*-*β*-Ionone		1489	-	0.27	0.39	0.16	-	-
48	*β*-Cadinene		1494	-	-	-	-	0.01	0.01
49	Bicyclogermacrene	C15′	1498	47.32	57.50	10.21	48.08	9.54	18.03
50	*α*-Muurolene		1502	-	-	-	-	0.07	-
51	*γ*-Cadinene		1517	-	-	-	-	0.25	0.32
52	*δ*-Cadinene	C16′	1527	0.10	-	-	-	0.91	1.32
53	Dihydroactinidiolide		1532	0.17	0.26	0.42	0.22	0.12	0.20
54	Cadina-1,4-diene		1536	-	-	-	-	-	0.05
55	Spathulenol		1580	-	-	-	-	0.34	-
56	Viridiflorol	C17′	1595	-	-	24.19	-	-	-
Total identified [%]				95.99	97.09	93.06	92.99	94.58	89.35

^1^ CD: Compound designation of the major components (average percentages ≥ 1.0% in at least one of the samples), which were included in the principal component analysis. ^2^ RI: Retention index determined relative to a homologous series of *n*-alkanes (C_9_–C_25_) on a HP-5MS column; ^3^ Tentatively identified.

**Table 3:** Composition (%) of major identified VOCs of *S. romana* and *S. montana* obtained by HS-SPME/GC-MS with DVB/CAR/PDMS and PDMS/DVB fiber.

No.	Compound	CD ^1^(A ^2^/B ^3^)	*S. romana* K (A ^2^/B ^3^)	*S. romana* B (A ^2^/B ^3^)	*S. romana* M (A ^2^/B ^3^)	*S. romana* P (A ^2^/B ^3^)	*S. montana* J (A ^2^/B ^3^)	*S. montana* M (A ^2^/B ^3^)
1	Acetic acid	C1/-	-/-	0.85/-	3.11/-	1.33/-	0.32/-	0.55/-
2	*α*-Thujene	C2/-	0.47/0.55	1.03/0.41	1.08/0.79	0.21/0.58	0.16/0.07	0.05/0.04
3	*α*-Pinene	C3/C1′	1.32/2.09	0.94/2.31	2.51/3.91	2.19/2.68	3.42/1.69	1.23/1.14
4	Benzaldehyde	C4/-	0.44/0.06	0.32/0.08	2.58/0.40	0.71/0.15	0.37/0.04	0.42/0.10
5	*β*-Pinene	C5/C2′	3.69/6.78	2.61/8.01	6.67/13.26	8.01/10.35	2.81/1.80	1.52/1.46
6	*β*-Myrcene	C6/-	0.80/0.55	0.84/0.40	1.27/0.99	0.88/0.80	0.91/0.28	0.64/0.20
7	*α*-Phellandrene	C7/-	0.32/0.15	3.37/0.83	2.54/0.27	0.20/0.22	0.10/0.02	0.09/-
8	*α*-Terpinene	C8/C3′	0.62/0.53	0.61/-	1.32/1.15	0.38/1.01	0.07/-	0.07/-
9	*p*-Cymene	C9/C4′	0.99/0.81	1.81/0.16	5.49/3.10	1.32/1.57	0.04/-	0.05/-
10	Limonene	C10/C5′	6.34/3.86	7.58/1.97	7.10/3.31	1.68/2.27	8.14/3.74	6.31/2.91
11	Benzyl alcohol	C11/C6′	2.96/0.63	3.82/0.96	13.28/2.55	3.84/1.12	0.84/0.16	2.14/0.55
12	*γ*-Terpinene	C12/C7′	1.75/2.53	0.98/0.11	3.67/8.96	1.40/4.49	0.08/-	0.08/-
13	Octan-1-ol	C13/C8′	1.37/1.17	2.22/1.33	3.55/2.46	2.50/2.24	0.35/0.12	0.29/0.16
14	*α*-Terpineol	C14/-	0.50/0.22	3.85/0.58	1.25/0.41	0.02/-	0.04/-	-/-
15	Myrtenal	C15/-	0.39/0.15	0.54/0.07	1.03/0.70	0.47/0.11	-/0.02	-/-
16	Bicycloelemene	-/C9′	0.75/2.18	0.53/2.40	-/0.43	0.40/1.73	0.22/0.34	0.41/0.68
17	*α*-Copaene	C16/C10′	0.18/0.07	-/-	-/-	-/-	1.67/1.33	2.02/1.55
18	*α*-Gurjunene	C17/-	1.17/-	0.95/-	0.61/0.79	0.85/-	0.12/-	0.41/-
19	Aristolene	C18/-	1.08/-	0.91/-	-/-	-/-	-/-	-/-
20	*trans*-Caryophyllene	C19/C11′	-/0.54	-/0.91	3.60/6.35	23.88/2.30	11.89/15.49	6.56/6.65
21	Aromadendrene	C20/-	1.11/0.25	1.05/-	-/-	0.96/-	0.23/-	2.87/-
22	Alloaromadendrene	C21/-	7.32/-	6.69/-	2.35/-	6.27/0.25	0.76/0.06	0.90/0.14
23	Selina-5,11-diene ^4^	C22/-	2.52/-	2.28/0.26	-/-	1.91/-	-/-	-/-
24	*trans*-*β*-Farnesene	C23/C12′	5.12/4.88	12.79/7.71	1.00/3.55	5.58/4.90	4.83/4.02	3.56/2.17
25	Isocaryophyllene	C24/C13′	4.70/7.10	2.39/6.85	-/-	3.22/5.17	-/-	-/-
26	*α*-Amorphene	C25/-	0.58/-	-/-	-/-	-/-	2.84/0.19	3.31/-
27	Germacrene D	C26/C14′	1.50/12.18	0.06/2.16	-/1.77	-/1.22	23.23/53.63	17.04/51.08
28	*β*-Cadinene	C27/-	2.19/-	1.68/-	-/-	1.24/-	0.98/0.01	1.47/0.01
29	Ledene	C28-/	-/-	-/-	8.20/-	-/-	2.27/-	-/-
30	Bicyclogermacrene	C29/C15′	23.04/47.32	20.48/57.50	-/10.21	15.16/48.08	4.19/9.54	11.76/18.03
31	*α*-Muurolene	C30/-	0.61/-	0.18/-	-/-	-/-	1.78/0.07	2.93/-
32	*γ*-Cadinene	C31/-	0.58/-	-/-	-/-	-/-	3.33/0.25	3.37/0.32
33	*δ*-Cadinene	C32/C16′	1.84/0.10	0.15/-	-/-	-/-	7.57/0.91	8.85/1.32
34	Cadina-1,4-diene	C33/-	0.20/-	-/-	-/-	-/-	0.94/-	1.24/0.05
35	*α*-Cadinene	C34/-	0.24/-	-/-	-/-	-/-	1.41/-	1.70/-
36	*α*-Calacorene	C35/-	0.47/-	0.04/-	-/-	-/-	1.05/-	1.36/-
37	Spathulenol	C36/-	0.58/-	1.30/-	-/-	0.52/-	0.15/0.34	0.47/-
38	Viridiflorol	C37/C17′	0.42/-	0.36/-	10.29/24.19	0.20/-	0.08/-	0.21/-

^1^ CD: Compound designation of the major components (average percentages ≥ 1.0% in at least one of the samples), which were included in the principal component analysis; ^2^ A: Compound extracted using DVB/CAR/PDMS fiber; ^3^ B: Compound extracted using PDMS/DVB fiber; ^4^ Tentatively identified.

**Table 4:** Sample codes, voucher numbers, harvesting locations, geographic coordinates and climatic affiliations according to Köppen climate classification for investigated *Sideritis* species.

Species	Sample Code	Voucher No.	Location	Date of Collection	Latitude	Longitude	Köppen Climate Classification ^1^
*S. romana*	*S. romana* K	18 021	Istria, Kamenjak	13 June 2020	44°47′46.15″ N	13°54′25.96″ E	Cfa ^2^
	*S. romana* B	18 023	Dalmatia, Blato	21 June 2020	43°10′12.71″ N	17°11′45.75″ E	Csa ^3^
	*S. romana* M	18 020	Dalmatia, Morinje Bay	7 June 2020	43°40′44.79″ N	15°57′41.25″ E	Csa ^3^
	*S. romana* P	18 022	Dalmatia, Brač, Pražnica	20 June 2020	43°19′1.3″ N	16°40′24.45″ E	Csb ^4^
*S. montana*	*S. montana* J	18 010	Dalmatia, Ježević	6 June 2020	43°55′1.51″ N	16°28′8.41″ E	Cfb ^5^
	*S. montana* M	18 011	Dalmatia, Mosor, Gornje Sitno	22 June 2020	43°31′13.56″ N	16°36′10.44″ E	Cfa ^2^

^1^ Data obtained from Šegota and Filipčić [45]; ^2^ Cfa: temperate humid climate with hot summer; ^3^ Csa: Mediterranean climate with hot summer; ^4^ Csb: Mediterranean climate with warm summer; ^5^ Cfb: temperate humid climate with warm summer.

## Data Availability

The data presented in this study are available in article.

## Sample Availability

Samples of the compounds are available from the authors.
